# Seeking Benefits or Avoiding Risks? Public Opinion Content and Consumers’ Purchase Intention Toward Aquatic Products: The Case of Basa Fish

**DOI:** 10.3390/foods15183169

**Published:** 2026-09-08

**Authors:** Xiaoyan Wang, Jianbo Luo, Yizhuo Zhang, Xiang Gao, Yongxiang Zhang, Yue Sun, Lejun Xu

**Affiliations:** 1 Chinese Academy of Fishery Sciences, No. 150 West Qingta Road, Fengtai District, Beijing 100141, China; 2 School of Accounting (Wealth Management), Ningbo University of Finance and Economics, No. 899 Xueyuan Road, Haishu District, Ningbo 315175, China

**Keywords:** aquatic products, public opinion, perceived benefits, perceived sacrifices, purchase intention, regulatory focus

## Abstract

Information asymmetry in aquatic product markets heightens consumers’ perceived food safety risks. Using basa fish as the focal product, this study examined how public opinion content influences purchase intention through perceived benefits and perceived sacrifices, and whether regulatory focus moderates these effects. Based on a scenario experiment, prior basa fish consumers were randomly assigned to positive, balanced, or negative video conditions; the final per-protocol sample comprised 742 participants. Baseline-adjusted analyses showed that positive content produced the highest perceived benefits and purchase intention and the lowest perceived sacrifices, balanced content produced intermediate responses, and negative content produced the least favorable responses. Significant indirect effects operated through perceived benefits and perceived sacrifices. Regulatory focus produced selective moderation: among more promotion-oriented consumers, the positive-versus-negative and balanced-versus-negative advantages in perceived benefits, and the balanced-versus-negative reduction in perceived sacrifices, were greater; among more prevention-oriented consumers, the balanced-versus-negative advantage in purchase intention was greater. These findings clarify how complete public opinion message packages shape consumer evaluations and provide guidance for aquatic product communication and market governance.

## 1. Introduction

Aquatic products serve as a major source of high-quality dietary protein and play a vital role in improving dietary quality [1,2]. As consumption patterns have evolved, public demand has shifted from simply meeting quantity needs to seeking higher quality, greater safety, and better nutrition. This shift reflects consumers’ increasing expectations of perceived benefits [3]. However, aquatic products are highly perishable and freshness-sensitive. Their production and distribution involve long value chains and multiple supply chain participants. These characteristics increase the likelihood of hidden quality and safety risks, including veterinary drug residues, illegal additives, and environmental contamination [4,5,6,7]. These characteristics also exacerbate information asymmetry in the marketplace. Consequently, consumers become more sensitive to potential risks and less tolerant of uncertainty. This response further increases their perceived sacrifices. Because consumers cannot directly evaluate product quality before purchase, they rely heavily on external cues when assessing risk and balancing perceived benefits against perceived sacrifices [8].

Online public opinion has become an important source of information about the safety and value of aquatic products [8]. Public opinion reflects the collective emotions, attitudes, and opinions of individuals and social groups within a given social context [9]. Public opinion content can be classified as positive, negative, or balanced (mixed-valence) according to its emotional valence. Positive content expresses praise, support, or affirmation. Negative content conveys criticism, doubt, or opposition. Balanced content presents both favorable and unfavorable statements without a clear overall emotional orientation [10]. Because online public opinion spreads rapidly and widely, its valence can shape consumer cognition and purchase decisions [11].

Previous studies generally show that positive information improves product evaluations, whereas negative information increases perceived risk [10,12]. Negative information may receive greater attention because consumers regard it as more diagnostic [10,13]. However, evidence concerning balanced valence information is mixed [13,14]. Negative cues embedded in mixed-valence content may be amplified by negativity bias [15,16]. Conversely, two-sided messages may enhance source credibility, although their persuasive effects depend on message structure and context [17]. This unresolved issue is especially relevant to aquatic products. Balanced content is common in news reports, safety notices, and online discussions, yet its effect on aquatic product consumption remains underexplored [18]. Moreover, most valence research concerns general consumer goods or ordinary agricultural products [19]. Its conclusions may not apply to aquatic products, which involve greater perceived risks and stronger dependence on external information.

After exposure to public opinion information, consumers’ future purchasing decisions undergo changes. This process simultaneously involves an overall evaluation by consumers based on what is received (perceived gain) and what is given up (perceived loss/sacrifice) [20]. Perceived benefits refer to the nutrition, health benefits, taste, and psychological satisfaction that consumers expect from aquatic products [20,21]. Perceived sacrifices include monetary costs and non-monetary costs. The latter include potential health risks, information-search costs, and food safety anxiety [22]. Public opinion content may change the relative importance of these benefits and sacrifices. Positive content may emphasize product value, whereas negative content may heighten safety concerns. Balanced content may draw attention to both sides and increase uncertainty.

Consumer responses may also differ according to individual traits, particularly when faced with different purchase goals. Regulatory focus describes how individuals pursue goals and respond to gains or losses, and is primarily categorized into promotion focus and prevention focus [23]. Promotion-focused consumers place greater emphasis on growth, advancement, and gains. Prevention-focused consumers are more concerned with safety, responsibility, and loss avoidance [24,25]. These orientations may influence which parts of public opinion content receive the most attention. They may also affect how consumers balance perceived benefits against perceived sacrifices. Regulatory focus has been examined in online-review and marketing-communication contexts [12,26]. This study extends that line of inquiry to aquatic product consumption.

Existing research therefore has several gaps. Most studies examine the evolution of public opinion events at the macro level or focus on general consumer goods [10,12,13,18]. Less attention has been paid to consumers’ cognitive responses in aquatic product markets. Research has also emphasized negative public opinion and comparisons between positive and negative content [10,12,27]. Balanced content remains underexplored, despite its prevalence in food-related communication [13,14,19]. Moreover, perceived benefits, perceived sacrifices, and regulatory focus have rarely been examined together. To address these gaps, this study uses a scenario-based experiment focused on basa fish to examine how positive, balanced, and negative public opinion content affect consumers’ purchase intention. It also investigates the roles of perceived benefits, perceived sacrifices, and regulatory focus in this process.

## 2. Theoretical Framework

### 2.1. Public Opinion Content

Consumers exhibit a pronounced negativity bias when processing information. They are more likely to attend to and share negative information than positive information. This pattern is consistent with the psychological principle that “bad is stronger than good” across emotional, attentional, and decision-making processes [15]. Public opinion dissemination is fundamentally a process of information transmission through which the emotional valence of information shapes consumers’ decisions and behaviors [28]. Aquatic products are highly perishable, and aquaculture environments are inherently uncertain. In addition, their supply chains are complex. These characteristics make consumers more sensitive to public opinion related to aquatic products [27].

Prior research indicates that positive online reviews may be perceived as more trustworthy, credible, and helpful than negative or two-sided reviews [12], while perceived review usefulness plays an important role in attitude and behavioral-intention formation [13]. Experimental research on fish-consumption communication further indicates that benefit-only messages can increase intended fish consumption [29]. In the present study, the positive public opinion content was operationalized by emphasizing affordable prices, high nutritional value, good taste, and convenience. Based on the preceding evidence, we expect this condition to increase consumers’ perceived benefits and purchase intention.

Experimental research on fish-consumption communication indicates that risk-only messages can worsen consumers’ health and safety evaluations and reduce intended fish consumption [29]. In the present study, the negative public opinion content was operationalized by emphasizing poor aquaculture conditions, parasite contamination, pesticide residues, excessive use of food additives, and unhygienic processing environments. Based on the preceding evidence, we expect this condition to increase consumers’ perceived health risks and perceived sacrifices, and thereby reduce purchase intention.

Mixed-valence reviews, which contain both positive and negative information, have an ambiguous influence on product sales [14]. However, online public opinion spreads rapidly across digital platforms. Consumers tend to pay greater attention to negative information because of negativity bias. Consequently, the influence of negative information embedded in mixed-valence public opinion content is amplified by consumers’ negativity bias. This amplification is more likely to evoke negative responses during subsequent purchase decisions.

Based on the above discussion, this study proposes the following hypotheses:

**H1.**  *Public opinion content has a significant impact on consumers’ perceived benefits, perceived sacrifices, and purchase intention.*

**H1a.**  *Compared with negative public opinion content, positive public opinion content is expected to produce higher perceived benefits and purchase intention and lower perceived sacrifices.*

**H1b.**  *Compared with negative public opinion content, balanced public opinion content is expected to produce higher perceived benefits and purchase intention and lower perceived sacrifices.*

**H1c.**  *Compared with balanced public opinion content, positive public opinion content is expected to produce higher perceived benefits and purchase intention and lower perceived sacrifices.*

### 2.2. Perceived Benefits and Perceived Sacrifices

According to perceived value theory, consumers form purchase intention toward basa fish by weighing the perceived benefits of its consumption against the associated perceived sacrifices [20]. These evaluations are shaped by positive or negative public opinion information. Specifically, public opinion information influences consumer decisions by shaping consumers’ subjective evaluations of product value [30]. Aquatic products are widely consumed because they are rich in micronutrients, high in protein, low in fat, easy to digest, and associated with health benefits [31]. However, purchasing aquatic products also requires consumers to pay monetary costs and accept potential quality and safety risks. Product defects or quality failures may also increase consumers’ time, financial, and psychological costs, thereby triggering negative emotions such as regret.

In the context of food consumption decisions, external information primarily influences consumers by shaping their perceived benefits and perceived sacrifices [32]. This cognitive evaluation subsequently shapes consumers’ purchase intention. Dodds et al. (1991) [33] demonstrated that external information does not influence purchase intention directly. Instead, its effect is mediated through perceived value. Positive public opinion enhances consumers’ perceived functional and experiential value by conveying favorable signals [20]. This process increases consumers’ perceived benefits. Negative reviews increase consumers’ perceived risk. This, in turn, increases perceived sacrifices and reduces purchase intention [16]. Mixed-valence evaluations simultaneously influence perceived benefits and perceived sacrifices. As a result, consumers experience greater uncertainty when making purchase decisions [34].

Based on the preceding arguments, this study proposes the following hypothesis:

**H2.**  *Perceived benefits and perceived sacrifices mediate the relationship between public opinion content and consumers’ purchase intention.*

### 2.3. Regulatory Focus

Grounded in regulatory focus theory, consumers with different regulatory orientations should assign different weights to the informational characteristics contained in public opinion messages [35,36,37]. Promotion-focused consumers pursue advancement and gains and should therefore attend more closely to gain-related product attributes, such as high-quality protein and nutritional value, taste, convenience, and affordability. Prevention-focused consumers prioritize safety, responsibility, and loss avoidance and should therefore attend more closely to risk and risk-control cues, such as aquaculture conditions, chemical treatments and additives, environmental hygiene, inspection, and assurances that food-safety hazards are controlled [38]. Regulatory focus is therefore expected to shape the relative weight assigned to these informational cues, rather than to produce a uniform response to message valence.

Based on this reasoning, this study proposes the following hypothesis:

**H3.**  *Regulatory focus moderates the effects of public opinion content on consumers’ perceived benefits, perceived sacrifices, and purchase intention.*

**Figure 1:**
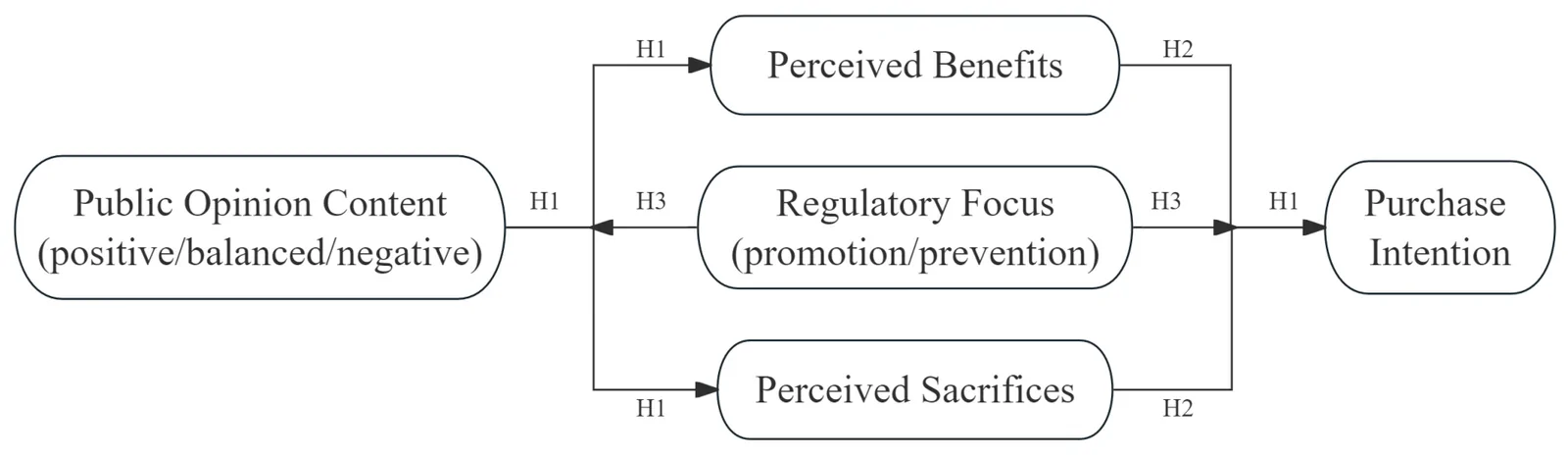
Conceptual framework linking public opinion content to purchase intention directly and indirectly through perceived benefits and perceived sacrifices, with regulatory focus as a moderator.

Accordingly, the conceptual framework of the study is presented in Figure 1.

## 3. Materials and Methods

This study adopted a scenario-based simulation experiment combined with a questionnaire survey to investigate the underlying mechanisms by which public opinion stimuli concerning aquatic products influence consumer purchase intentions. The experimental stimuli focused on public opinion incidents involving aquatic products that received substantial public attention and evoked strong consumer concern. The stimuli were constructed from real-world events to enhance ecological validity and encourage participants to make decisions that more closely reflected real consumption contexts. Public opinion stimuli were presented as short videos, reflecting the widespread use of this information format [39]. The measurement items were adapted from well-established and validated scales. The items were further refined to fit the context of public opinion concerning aquatic products.

The questionnaire comprised three primary sections. The first section was designed as a screening process to assess participant eligibility. Specifically, participants were screened to confirm that they were active consumers of aquatic products. This procedure minimized the possibility that unfamiliarity with aquatic products would confound the measurement of regulatory focus. Participants first completed the regulatory focus scale. Baseline perceived benefits, perceived sacrifices, and purchase intention were then measured before exposure to the experimental stimuli. The second section contained the scenario-based experiment. Participants were randomly assigned to one of the experimental stimuli representing different public opinion stimuli. After exposure to the stimuli, participants’ perceived benefits, perceived sacrifices, and purchase intention were measured. The third section collected participants’ demographic information.

### 3.1. Variables Measurement

Purchase intention was measured using a 5-point Likert scale adapted from Dodds et al. (1991) [33]. Responses ranged from 1 (“strongly disagree”) to 5 (“strongly agree”). The identical item and response scale were administered before and after exposure to the experimental video. This format was retained because it is straightforward and respondent-friendly, facilitates rapid judgments based on participants’ immediate reactions, and ensures direct comparability between the pre- and post-exposure measurements. The item was intended to capture participants’ immediate stated willingness to purchase the product rather than to distinguish finely among different subjective purchase probabilities. The single-item operationalization was selected because the focal outcome was narrowly defined as willingness to purchase a specific and familiar product. This outcome was treated as a concrete, narrowly bounded judgment rather than as a comprehensive measure of the broader purchase-intention domain. Previous measurement research suggests that a single-item measure can be methodologically defensible when the focal construct consists of a concrete object evaluated on a concrete, unidimensional attribute [40,41]. The use of identical wording at both measurement occasions also ensured direct pre–post comparability and limited redundant repeated questioning in the short experimental procedure.

Perceived benefits and perceived sacrifices were based on scales adapted from Monroe and Krishnan (1985) [42] and Cox (1967) [43]. The measures were further contextualized for basa fish consumption. Perceived benefits were measured as an overall favorable appraisal using three items concerning preparation convenience, satisfaction derived from eating fish, and value for money. Perceived sacrifices were measured as an overall unfavorable appraisal using three items concerning potential harm to physical health, unfavorable evaluation by family or friends, and anticipated problems or regret. Regulatory focus was measured using a scale adapted from Higgins et al. (2001) [44]. The scale comprised six promotion-focus items and four prevention-focus items. Promotion-focus and prevention-focus scores were calculated separately as the unweighted means of their respective items. All constructs were measured using 5-point Likert scales. The corresponding measurement items are presented in Table 1.

### 3.2. Scenario-Based Experiment

The study was designed to examine the direct impact of public opinion content on consumers’ purchase intentions, as well as the underlying psychological mechanisms linking public opinion content to purchase intention through perceived benefits and perceived sacrifices. Public opinion stimuli concerning basa fish were developed because basa fish is one of China’s most heavily imported freshwater fish. It has also frequently been associated with negative public opinion controversies, such as “being marketed as sole fish,” “chemical soaking,” “low quality at low prices,” and “poor aquaculture environments.”

Drawing on real-world consumer evaluations of aquatic products, the public opinion stimuli were developed to represent three types of public opinion content. These stimuli were constructed around six content domains used to construct the experimental videos: purchase price, convenience of preparation, taste and flavor, nutritional value, health and safety, and environmental hygiene. To minimize the potential confounding effects of information volume and message structure on purchase intention, all public opinion stimuli were standardized in terms of word count and structural format.

#### 3.2.1. Experimental Design

This study adopted a single-factor between-subjects experimental design in which participants were randomly assigned to one of three complete public opinion video packages: positive, balanced, or negative. Although all videos drew on the same six construction domains, the individual claims were not matched item for item; evaluative direction and substantive attribute emphasis therefore varied together. The design compares complete message packages rather than isolating emotional valence. Price, convenience, taste and flavor, nutritional value, health and safety, and environmental hygiene were the six content domains used to construct the experimental videos. The study was designed to compare three active public opinion communication packages rather than exposure with no exposure. A valence-free product message could still be interpreted favorably or unfavorably according to participants’ prior experience, whereas a no-information condition would address a different research question and would not match the three video conditions in information exposure and task engagement. The design therefore estimates relative differences among active message packages.

**Figure 2:**
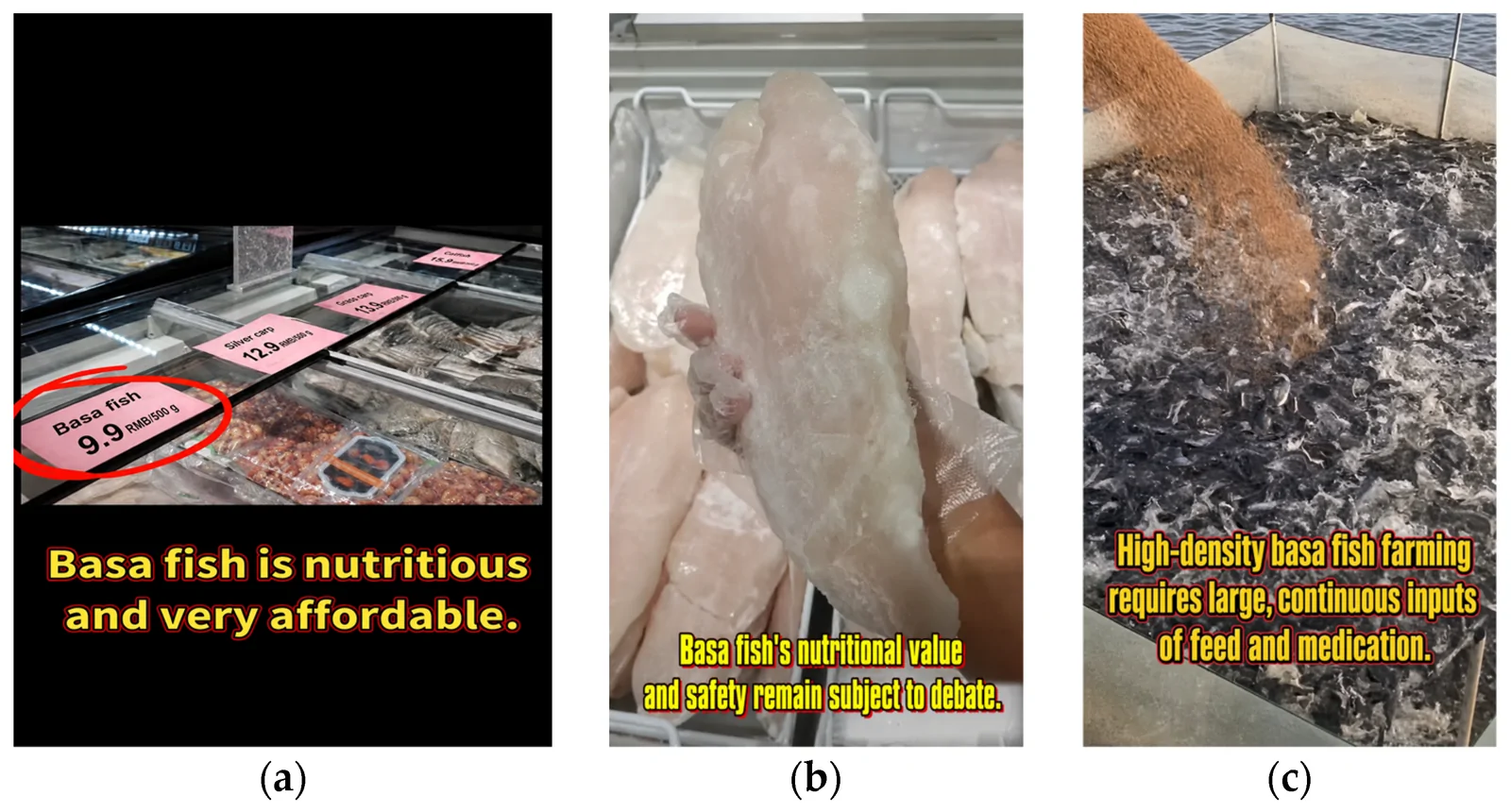
Representative stills from the public opinion video stimuli: (**a**) positive content highlighting the nutritional value and affordability of basa fish; (**b**) balanced content presenting favorable and unfavorable information about its nutritional value and safety; and (**c**) negative content depicting high-density farming and emphasizing intensive inputs of feed and medication. Participants viewed the complete original Chinese-language videos; these stills are presented only to illustrate the visual content of the three experimental conditions.

The positive condition featured affirmative and encouraging language that conveyed a clear supportive stance. The stimuli emphasized perceived benefits, including “rich in high-quality protein and unsaturated fatty acids,” “tender and boneless meat,” and “affordable prices.” The negative condition featured critical and cautionary language that conveyed a clear opposing or boycotting stance. The stimuli highlighted perceived sacrifices by describing potential risks, including “extremely high farming density,” “continuous chemical treatments,” and “artificial additives.” The balanced condition simultaneously presented positive and negative information stimuli. The stimuli maintained an objective and balanced tone without providing any purchase recommendations. Specifically, the stimuli first described perceived benefits, such as low prices, tender meat, and convenient preparation, and then introduced perceived sacrifices, including “limited nutritional value” and “concerns about farming practices and drug use.” Mixed-valence expressions, such as “somewhat controversial” and “a cautious attitude,” were incorporated to reinforce the objective tone. Sample video screenshots of the public opinion experimental stimuli are presented in Figure 2.

The experimental stimuli were developed from authentic media reports and presented as short videos. This presentation format was intended to activate consumers’ prior knowledge and experiences, thereby enhancing experimental realism and ecological validity [45]. The three continuous videos shared the same focal product, short-video format, broad sequence, approximate duration, audio presentation, and six stimulus-construction domains: price, preparation convenience, taste and texture, nutritional value, health and safety, and farming/environmental hygiene. The positive package emphasized product advantages, the negative package emphasized safety and production concerns, and the balanced package combined favorable and unfavorable cues. This design reflects the way real online public opinion is naturally bundled. It provides ecological validity for comparing complete communication packages.

#### 3.2.2. Pre-Test

The pilot test was conducted to evaluate potential biases associated with the wording of the public opinion stimuli. Following consultation with domain experts, the experimental stimuli were finalized. To verify the effectiveness of the experimental manipulation, participants were instructed to classify the public opinion content after reading the experimental stimuli. During the pre-test for Experiment 1, 250 questionnaires were distributed, and 225 valid responses were obtained. The agreement between participants’ classifications and the intended experimental design was 90%, indicating a high level of classification accuracy.

#### 3.2.3. Main Experiment

The main experiment was conducted between June and August 2025. In collaboration with a professional online survey agency (Credamo), participants were randomly recruited to complete the questionnaire. First, participants completed the adapted regulatory focus scale. They then completed the same brief baseline block in the following order: purchase intention, perceived benefits, and perceived sacrifices. Random assignment and stimulus exposure occurred only after completion of this block. Second, participants were randomly assigned to one of the public opinion stimuli concerning the target product. Third, participants completed the post-test by reassessing their perceived benefits, perceived sacrifices, and purchase intention toward the target product. Finally, demographic information was collected. After completing the experiment, participants received RMB 9. The amount was set with reference to compensation commonly used for local online scenario surveys of comparable length and task burden. The questionnaire contained 22 questions across three parts: screening and baseline measurement, the experiment and post-exposure measurement, and participant characteristics.

To improve data quality, viewing compliance was monitored as follows. Participants were required to manually initiate the assigned video and, after viewing it in full, manually close the video window before continuing with the questionnaire. Participants could stop viewing and withdraw from the survey at any time if the video caused discomfort. If they closed the video before completion, they could not access the post-video questions unless they reopened and completed the video. Skipping and fast-forwarding were disabled, whereas replaying was permitted so that participants could review information they might have missed. Each video included an enabled audio track and on-screen subtitles presenting the same information. The survey was completed predominantly on smartphones, with fewer than ten participants using computers. Participants were instructed to complete the pre- and post-exposure measures in the same setting. After viewing the stimulus, participants completed the condition-specific Yes/No manipulation-check item. A response was classified as a manipulation-check mismatch when the selected category did not correspond to the randomly assigned condition; such cases were excluded from the per-protocol analysis.

- Target Respondent Identification.

A screening question (“Have you ever purchased or consumed Basa fish?”) was administered before the formal questionnaire. Eligible respondents were adults aged 18 years or older with prior purchase or consumption experience with basa fish.

- Response Quality Control.

All questions, except for the screening item, were set as required. Participants could submit the survey only after completing all questions. In addition, a minimum response time was specified for each scenario. This procedure helped ensure that participants spent sufficient time reading the simulated public opinion stimuli before making their responses.

- Manipulation Checks.

Manipulation-check questions were embedded immediately after each scenario. Following the positive public opinion stimulus, participants answered the following single-choice question: “Do you think the video’s description of Basa fish is positive?” Participants in the negative condition were asked, “Do you think the above video’s description of Basa fish is negative?” Participants in the balanced condition were asked,” Do you think the above video’s description of Basa fish contains both positive and negative information?” Each item used Yes/No response options.

The participant flow was recorded sequentially. A total of 849 individuals entered eligibility screening. Twenty-eight individuals who reported no prior purchase or consumption experience with basa fish ended the survey before random assignment, leaving 821 eligible participants. These 821 participants were randomly assigned to the positive, balanced, or negative condition. Seven participants withdrew during video viewing and therefore did not proceed to the post-video measures, leaving 814 participants who completed the video and reached the manipulation check. Among these 814 participants, the manipulation-check response did not match the assigned condition for 19 of 266 participants in the positive condition (7.14%), 33 of 280 in the balanced condition (11.79%), and 20 of 268 in the negative condition (7.46%). There was no statistically significant evidence that the mismatch rates differed across the three conditions (χ^2^(2, N = 814) = 4.59, *p* = 0.101, Cramér’s V = 0.075), although the rate was numerically higher in the balanced condition. Excluding the 72 mismatched cases yielded a final per-protocol sample of 742 participants (positive: n = 247; balanced: n = 247; negative: n = 248), representing 90.38% of the randomized sample and 87.40% of those who entered eligibility screening.

### 3.3. Data Analysis

All primary outcome, mediation, and moderation models were estimated using the final per-protocol sample (N = 742). Participant characteristics were summarized using frequencies and percentages, and manipulation-check mismatch rates were compared across conditions using Pearson’s χ^2^ test with Cramér’s V. SPSS 23.0 was used for KMO and Bartlett’s tests, exploratory PCA, and Cronbach’s α. AMOS 23.0 was used to obtain the standardized CFA loadings, composite reliability, and average variance extracted. Robust maximum-likelihood CFA fit indices, Pearson HTMT with 5000 bootstrap resamples, and the outcome analyses were conducted in R 4.6.1. The factor structure was specified a priori on the basis of the theoretical model and adapted scales. Exploratory PCA and CFA were conducted on the same final sample (N = 742). PCA-derived weights were not used to construct the variables entered in the outcome models. Instead, these variables were calculated as unweighted means of their prespecified items, preserving their five-point interpretation. For perceived benefits and perceived sacrifices, the reliability and validity analyses used the post-exposure items. CFA fit was evaluated jointly using scaled χ^2^, robust CFI, robust TLI, robust RMSEA with its 90% CI, and SRMR [46]. Purchase intention was treated as a single observed item and was not included in internal-consistency, composite-reliability, AVE, or factor-loading assessments.

For H1, each post-exposure outcome was analyzed by baseline-adjusted ANCOVA with condition, the corresponding baseline score, gender, age, education, occupation, annual income, household composition, primary purchase channel, consumption frequency, proximity to water, self-rated health, and prior seafood-safety experience. The negative content condition was used as the active reference because the practical objective was to assess whether positive or balanced communication could improve consumer evaluations relative to adverse public opinion content. It was an active comparator rather than an untreated control. All three Holm-adjusted pairwise comparisons were reported, so the substantive conclusions did not depend on the choice of reference category. Type-II omnibus tests with HC3-robust standard errors, partial η^2^, adjusted means, 95% confidence intervals, and Holm-adjusted pairwise comparisons were reported. As a pre–post sensitivity specification, we estimated:

(1) Y_it_ = β_0_ + β_1_Post__t_ + β_2_Positive__i_ + β_3_Balanced__i_ + β_4_(Post__t_ × Positive__i_) + β_5_(Post__t_ × Balanced__i_) + γ′X__i_ + u_i_ + ε__it_

Post was coded 0 at pre-test and 1 at post-test. Positive and balanced were binary indicators coded 1 for participants assigned to the corresponding condition and 0 otherwise; participants in the negative condition therefore had positive = 0 and balanced = 0. Thus, the omitted cell was the negative condition at pre-test. In Equation (1), Y_it_ denotes the outcome for participant i at time t, X_i_ denotes the covariates listed above, u_i_ denotes the participant-specific random intercept, and ε_it_ denotes the residual. The interaction coefficients represent differential pre–post changes relative to the active negative reference.

For H2, the positive and balanced conditions were each contrasted with the active negative-content reference in a simultaneous parallel-mediation model that included post-exposure perceived benefits and perceived sacrifices, while controlling for both mediator baselines, baseline purchase intention, and the same covariates. Indirect effects, direct effects, total effects, and the difference between the two indirect pathways were estimated with 5000 nonparametric bootstrap resamples and percentile 95% confidence intervals, following contemporary multiple-mediator recommendations [47].

For H3, moderation was examined using three baseline-adjusted OLS models with post-exposure perceived benefits, perceived sacrifices, and purchase intention as the respective dependent variables. Each model included the corresponding baseline outcome. Promotion-focus and prevention-focus scores were mean-centered and entered together. Positive and balanced were condition indicators, with the negative-content condition as the active reference. Each model included the condition main effects, both regulatory-focus main effects, the four condition-by-regulatory-focus interaction terms, and the same covariates used in the H1 models. HC3 heteroskedasticity-robust standard errors were used. For each outcome, a robust Wald F test jointly evaluated the four interaction terms. The 12 coefficient-level interaction *p* values were adjusted using the Benjamini–Hochberg false-discovery-rate procedure.

## 4. Results

### 4.1. Descriptive Statistics

Table 2 describes the analyzed consumer sample and the covariates used in the adjusted models. Female respondents accounted for the majority of the sample. Most respondents were between 21 and 40 years old, representing the primary consumer group for aquatic products. A share of 96.49% of the sample had a junior-college qualification or higher. This composition is substantively aligned with the study context: people in this age range are typically independent consumers and intensive users of online information, social media, restaurant services, and e-commerce, where basa fish public opinion is commonly encountered.

Corporate employees and public servants constituted the largest occupational groups. These respondents generally had stable incomes and were highly exposed to internet- and social media-based information in their daily lives. The sample was predominantly composed of middle-income consumers, with nearly 50% reporting an annual income between RMB 120,000 and RMB 240,000. This income group is likely to place greater emphasis on product quality and safety, thereby enhancing their perceived benefits from aquatic product consumption. In addition, nearly 80% of the respondents reported living in households with elderly family members or young children. These households are generally more sensitive to food safety issues, suggesting greater concern about potential health-related perceived sacrifices. The distribution of respondents across coastal and non-coastal regions was relatively balanced. Fresh food supermarkets were the most frequently selected primary purchase channel, followed by restaurants/cafeterias and farmers’ markets/seafood markets. More than 90% of the respondents reported consuming aquatic products regularly or occasionally. This finding indicates widespread consumer acceptance of aquatic products. Moreover, 95.42% of the respondents rated their health status as good or excellent. Nearly 90% of the respondents were aware of, or had heard about, aquatic product safety incidents. This finding suggests that most consumers possess a basic awareness of potential risks associated with aquatic products.

### 4.2. Reliability and Validity Analysis

Bartlett’s test of sphericity for the overall scale was significant (*p* < 0.001), and the Kaiser–Meyer–Olkin (KMO) value was 0.90, indicating that the data were suitable for factor analysis [48]. As shown in Table 3, the standardized factor loadings for all measurement items were greater than 0.69, indicating good indicator reliability. Cronbach’s alpha and composite reliability (CR) values exceeded 0.85 for all multi-item constructs. The average variance extracted (AVE) for each multi-item construct exceeded 0.50.

Table 4 presents the discriminant validity results for promotion focus, prevention focus, perceived sacrifices, and perceived benefits. The square root of the AVE for each construct was greater than its correlations with the other constructs, supporting satisfactory discriminant validity. In addition, the HTMT ratio between perceived benefits and perceived sacrifices was 0.8936. Its 95% bootstrap confidence interval, based on 5000 resamples, did not include 1.00, 95% CI [0.8667, 0.9185], supporting the empirical distinctiveness of the two constructs under the HTMT inference criterion [49]. Because the point estimate was close to the 0.90 benchmark, we acknowledge that their discriminant validity is borderline. Nevertheless, the well-fitting two-factor model and its substantial improvement over the one-factor model support retaining the theoretically distinct constructs. The two-factor perceived benefit/perceived sacrifice model fit well: scaled χ^2^(8) = 14.35, *p* = 0.073, robust CFI = 0.998, robust TLI = 0.997, robust RMSEA = 0.035, 90% CI [0.000, 0.063], and SRMR = 0.008.

### 4.3. Hypothesis Testing

#### 4.3.1. Baseline-Adjusted Effects of Public Opinion Content

Condition had significant baseline-adjusted effects on perceived benefits, F(2, 705) = 918.06, *p* < 0.001, partial η^2^ = 0.723; perceived sacrifices, F(2, 705) = 1222.02, *p* < 0.001, partial η^2^ = 0.776; and purchase intention, F(2, 705) = 820.30, *p* < 0.001, partial η^2^ = 0.699. Adjusted means (negative, positive, balanced) were 2.17, 4.53, and 4.29 for benefits; 4.34, 1.67, and 2.45 for sacrifices; and 1.86, 4.45, and 3.97 for purchase intention. Every Holm-adjusted comparison was significant at *p* < 0.001 (Table 5). Because purchase intention was measured on a bounded five-point scale and baseline scores were relatively high, the purchase-intention results were interpreted primarily as baseline-adjusted differences among the three assigned video conditions within the retained per-protocol sample. The baseline-adjusted condition effect was statistically significant, indicating that the three conditions were associated with clearly different post-exposure purchase-intention levels after accounting for baseline purchase intention and the prespecified covariates. Time-by-condition linear mixed-model sensitivity analyses yielded the same directions and substantive conclusions as the baseline-adjusted ANCOVA. H1a–H1c were supported.

Relative to negative content, positive content increased adjusted benefits by 2.367 (95% CI [2.233, 2.501]) and purchase intention by 2.587 [2.428, 2.746], while reducing sacrifices by 2.666 [−2.796, −2.537]. Balanced content showed the same pattern: 2.125 [1.980, 2.270], 2.107 [1.939, 2.274], and −1.885 [−2.061, −1.709]. Positive content also outperformed balanced content for benefits (0.242 [0.139, 0.344]) and purchase intention (0.480 [0.322, 0.638]) and produced lower sacrifices (−0.781 [−0.943, −0.619]).

#### 4.3.2. The Mediating Role of Perceived Benefits and Perceived Sacrifices

Bootstrap results supported both proposed pathways (Table 6). For positive versus negative content, the indirect effect through benefits was 0.823 (95% bootstrap CI [0.579, 1.081]) and through sacrifices was 1.016 [0.738, 1.311]; the total indirect effect was 1.839 [1.579, 2.096]. For balanced versus negative content, the corresponding effects were 0.739 [0.523, 0.964], 0.719 [0.518, 0.933], and 1.458 [1.259, 1.657]. Direct effects remained positive: 0.752 [0.488, 1.043] and 0.645 [0.409, 0.897]. Neither contrast between pathway magnitudes was significant. H2 was supported as a statistical mediation pattern.

#### 4.3.3. Moderating Effects of Regulatory Focus

The four condition-by-regulatory-focus interactions were jointly significant for perceived benefits, F(4, 699) = 6.26, *p* < 0.001; perceived sacrifices, F(4, 699) = 6.09, *p* < 0.001; and purchase intention, F(4, 699) = 3.67, *p* = 0.006 (Table 7). After Benjamini–Hochberg correction across the twelve interaction tests, four interactions remained significant. Among more promotion-oriented consumers, the positive-versus-negative and balanced-versus-negative advantages in perceived benefits were greater (b = 0.273, HC3 SE = 0.092, q = 0.013; b = 0.320, HC3 SE = 0.098, q = 0.007). The reduction in perceived sacrifices under balanced rather than negative content was also greater among these consumers (b = −0.599, HC3 SE = 0.129, q < 0.001). Among more prevention-oriented consumers, balanced content had a greater purchase-intention advantage over negative content (b = 0.278, HC3 SE = 0.114, q = 0.046). The remaining eight interactions did not remain significant after FDR correction. H3 was therefore partially supported.

## 5. Discussion

This study used scenario-based simulation experiments to examine the underlying psychological mechanisms through which public opinion content influences consumer responses. Specifically, it investigated how public opinion content influences consumers’ perceived benefits, perceived sacrifices, and purchase intention. The findings are discussed below.

### 5.1. Public Opinion Content Produces an Ordered Pattern of Consumer Responses

The baseline-adjusted results revealed a consistent ordering across the three public opinion conditions. Positive content produced the highest perceived benefits and purchase intention and the lowest perceived sacrifices. Balanced content produced more favorable evaluations than negative content but did not achieve the same effects as positive content. Thus, balanced content was not simply equivalent to positive content with a more moderate tone. The coexistence of favorable and unfavorable cues created a distinct information environment in which the advantages of basa fish remained visible, while risk-related information continued to restrain consumer responses.

This pattern is consistent with loss-aversion and negativity-bias arguments. When favorable and unfavorable information is presented together, consumers may assign greater decision weight to potential losses and safety concerns than to benefits of a comparable magnitude [14,15]. Ambiguity and uncertainty processing provide a complementary explanation. Mixed information requires consumers to reconcile competing cues and may therefore encourage more cautious judgments than uniformly positive information. These mechanisms are particularly relevant to aquatic products because consumers cannot readily verify freshness, production conditions, residues, or other safety attributes before purchase. In this setting, even limited unfavorable information may remain highly salient. The present findings therefore extend loss-aversion, negativity-bias, ambiguity, and uncertainty-processing arguments to an aquatic product context characterized by information asymmetry and safety sensitivity.

### 5.2. Perceived Benefits and Perceived Sacrifices Statistically Mediate the Relationship Between Public Opinion Message Packages and Purchase Intention

The mediation analyses identified significant indirect effects through perceived benefits and perceived sacrifices. These findings support two complementary cognitive-evaluation pathways linking the public opinion message packages to purchase intention and extend perceived value theory to aquatic product public opinion communication.

The condition contrasts were associated with purchase intention both directly and indirectly through perceived benefits and perceived sacrifices. The significant indirect effects are consistent with two complementary cognitive-evaluation pathways. One pathway enhanced consumers’ recognition of the functional value and consumption experience associated with aquatic products, thereby increasing perceived benefits. The other pathway reduced consumers’ concerns about food safety and health risks, thereby decreasing perceived sacrifices. These findings are consistent with the trade-off framework between perceived benefits and perceived sacrifices proposed by Zeithaml (1988) [20]. They also extend the logic of loss aversion derived from prospect theory to the context of aquatic product consumption. Aquatic products provide high-quality protein and essential micronutrients. They also offer desirable sensory attributes and strong satiety, all of which contribute to consumers’ perceived benefits. By contrast, concerns about drug residues, food additives, and parasites represent important sources of perceived sacrifices. When consumers are exposed to public opinion content about aquatic products, they do not passively receive information. Instead, they actively evaluate potential benefits and sacrifices before forming purchase intention.

Together, the significant indirect effects show that both benefit-related and sacrifice-related evaluations are relevant to consumers’ purchase-intention responses to public opinion messages. This dual-path pattern extends perceived value theory to the communication of food-safety information about aquatic products and supports communication strategies that address product value and safety-related concerns together. According to perceived value theory, the positive and negative information embedded in public opinion stimuli directly shapes consumers’ evaluations of the value of purchasing aquatic products. Therefore, the effectiveness of public opinion management depends not only on the information itself but also on its ability to influence consumers’ cognitive evaluation of perceived benefits and perceived sacrifices. This cognitive process ultimately shapes purchase intention.

### 5.3. Regulatory Focus Moderates Consumer Responses to Public Opinion Content

The results show that consumers’ regulatory orientation changed how they evaluated the three public opinion messages. For more promotion-oriented consumers, positive and balanced content had larger advantages over negative content in perceived benefits. Balanced content also reduced perceived sacrifices more strongly relative to negative content. Promotion-oriented individuals tend to pursue gains and advancement, so they are more likely to notice nutritional value, taste, convenience, affordability, and other favorable attributes. Even when balanced content included unfavorable cues, the presence of recognizable benefits gave these consumers a stronger basis for evaluating what they could gain from purchasing basa fish. For more prevention-oriented consumers, balanced content had a larger advantage over negative content in purchase intention. Prevention-oriented individuals are more concerned with safety and avoiding losses. The balanced message acknowledged unfavorable information while also presenting favorable information, whereas the negative message offered only adverse cues. This combination may have allowed prevention-oriented consumers to consider the identified risks without allowing their purchase decisions to be determined solely by negative information.

These findings are consistent with regulatory focus theory, which proposes that promotion-oriented consumers give greater weight to possible gains, whereas prevention-oriented consumers give greater weight to safety and loss avoidance [35,36,37,38]. Regulatory orientation did not simply make consumers more or less responsive to every message. Instead, it changed which part of the information played a greater role in their judgment. Promotion orientation was reflected mainly in consumers’ evaluations of benefits and sacrifices, whereas prevention orientation was reflected in purchase intention when balanced information was compared with wholly negative information. This pattern helps explain why consumers exposed to the same public opinion message may reach different judgments: they focus on different aspects of the information when evaluating an aquatic product.

## 6. Conclusions

### 6.1. Theoretical Contributions

This study makes three bounded contributions. First, it applies a dual-path perceived-value framework to online communication about basa fish. Second, it shows that balanced, mixed-valence communication occupies an intermediate position and extends loss-aversion, ambiguity, and uncertainty-processing arguments to an aquatic product context. Third, it extends regulatory-focus theory to aquatic product public opinion communication by showing that promotion-oriented consumers respond more strongly to benefit and sacrifice differences between message conditions, whereas prevention-oriented consumers show a stronger purchase-intention response to balanced rather than wholly negative information.

### 6.2. Managerial Implications

Public communication about aquatic products should provide verified information about product benefits while addressing safety concerns clearly and directly. When favorable and unfavorable information is presented together, communicators should distinguish established facts, remaining uncertainty, and corrective actions to reduce ambiguity. The moderation patterns also suggest that audience regulatory orientation may be considered when testing targeted communication strategies.

### 6.3. Limitations and Future Directions

This study has several limitations. First, the online survey experiment simulated consumer decision-making and may not fully capture actual consumption environments. Second, the findings are based on a per-protocol sample of adult online consumers with prior basa fish experience, concentrated among respondents aged 21–40 years and those with a college education, which limits population-level generalizability. Third, the videos bundled evaluative direction with ecologically typical attributes; therefore, the estimated condition contrasts concern complete message packages rather than pure valence. Fourth, although the brief baseline block and question ordering may have reduced mediator-related priming, they could not eliminate broader pre-test sensitization. Condition-specific manipulation-check mismatch rates did not differ significantly, but the primary analyses remained per-protocol; a full intention-to-treat analysis was not conducted. Fifth, the strong association between perceived benefits and perceived sacrifices, together with the nonrandomized mediator–outcome paths, limits exclusive causal-mediation claims. Finally, purchase intention was measured with a single self-reported item, which does not permit assessment of internal-consistency reliability and may be susceptible to item-specific measurement error.

Future research should evaluate public opinion stimuli in actual consumption settings and use preregistered factorial designs that match attribute content across valence conditions. It should also retain complete randomized follow-up, experimentally manipulate the proposed mediators, include behavioral outcomes, and recruit samples suitable for broader population inference. Purchase intention should be assessed using multiple nonredundant items covering willingness to try, purchase likelihood, purchase consideration, purchase willingness, and recommendation intention.

## Figures and Tables

**Table 1:** Measures, item codes, item wording, and sources for purchase intention, regulatory focus, perceived benefits, and perceived sacrifices in the basa fish experiment.

Variables	Code	Measurement Items	Sources
Purchase Intention	GM	I am willing to purchase Basa fish.	Dodds et al. (1991) [33]
Promotion Focus	CJ1	Compared with most people, I am typically unable to obtain what I desire.	Higgins et al. (2001) [44]
	CJ2	I have very few hobbies or activities that interest me or motivate me to invest effort.	
	CJ3	I often accomplish things that excite me and motivate me to work harder.	
	CJ4	When it comes to matters that are important to me, I find that I do not perform as well as I would like.	
	CJ5	When I try something new, I generally perform well.	
	CJ6	I feel that I have achieved some success in life.	
Prevention Focus	YF1	Growing up, I often crossed limits my parents would tolerate.	
	YF2	Growing up, I often made my parents angry.	
	YF3	Growing up, I often did things my parents disapproved of.	
	YF4	Growing up, I usually followed the rules established by my parents.	
Perceived Sacrifices	FX1	My physical health could be negatively affected.	Monroe and Krishnan (1985) [42]; Cox (1967) [43]
	FX2	My family or friends would consider my decision unwise.	
	FX3	I might encounter problems and regret my decision later.	
Perceived Benefits	JZ1	The convenience of preparing this product makes me feel at ease.	
	JZ2	It satisfies my desire to eat fish.	
	JZ3	It offers good value for money.	

**Table 2:** Demographic and consumption characteristics of the analyzed nationwide online sample of consumers with prior basa fish purchase or consumption experience. Percentages are calculated within the full analyzed sample (N = 742).

Variables	Category	Frequency	Percentage (%)
Gender	Male	312	42.05
	Female	430	57.95
Age	18–20	19	2.56
	21–30	337	45.42
	31–40	321	43.26
	41–50	43	5.80
	51–60	18	2.43
	61 and above	4	0.54
Education	Middle school or below	5	0.67
	High school or technical secondary school	21	2.83
	Bachelor or junior college	558	75.20
	Master and above	158	21.29
Occupation	Government or public institution staff	82	11.05
	Self-employed	20	2.70
	Company employee	517	69.68
	Student	76	10.24
	Agriculture/forestry/fishing/farming worker	5	0.67
	Freelancer	21	2.83
	Retired	3	0.40
	Other	18	2.43
Annual income (CNY)	Below 60,000	48	6.47
	60,000–120,000	168	22.64
	120,000–240,000	323	43.53
	240,000–600,000	192	25.88
	600,000 and above	11	1.48
Presence of children aged ≤ 6 or elderly aged ≥ 60 in household	Yes	591	79.65
	No	151	20.35
Primary purchase channel	Restaurant/cafeteria	184	24.80
	Fresh food supermarket	324	43.67
	Online platform	62	8.36
	Farmers’ market/seafood market	172	23.18
Consumption frequency	Rarely or never	48	6.47
	Occasionally	452	60.92
	Often	234	31.54
	Very frequently	8	1.08
Residence near rivers, lakes, or seas	Yes	382	51.48
	No	360	48.52
Health status	Excellent	346	46.63
	Good	362	48.79
	Fair	31	4.18
	Poor	3	0.40
Experience of aquatic product safety incidents	Never	79	10.65
	Heard or know about	607	81.81
	Experienced by self or relatives/friends	56	7.55

**Table 3:** Standardized confirmatory factor analysis loadings, Cronbach’s α, composite reliability, and average variance extracted for promotion focus, prevention focus, and the post-exposure perceived benefit and perceived sacrifice measures (N = 742).

Variable	Code	Standard Factor Loadings	Cronbach’s α	AVE	Composite Reliability
Promotion Focus	CJ1	0.739	0.8751	0.5434	0.8771
	CJ2	0.7365			
	CJ3	0.7159			
	CJ4	0.7733			
	CJ5	0.7357			
	CJ6	0.7215			
Prevention Focus	YF1	0.7762	0.8502	0.5962	0.8547
	YF2	0.6986			
	YF3	0.8113			
	YF4	0.7975			
Perceived Sacrifices	FX1	0.9326	0.9465	0.8561	0.9469
	FX2	0.9016			
	FX3	0.9411			
Perceived Benefits	JZ1	0.8770	0.9188	0.7948	0.9207
	JZ2	0.9240			
	JZ3	0.8726			

**Table 4:** Inter-construct correlations and square roots of average variance extracted for promotion focus, prevention focus, post-exposure perceived sacrifices, and post-exposure perceived benefits (N = 742).

Variables	Promotion Focus	Prevention Focus	Perceived Sacrifices	Perceived Benefits
Promotion Focus	0.7372			
Prevention Focus	0.3640	0.7721		
Perceived Sacrifices	−0.1895	−0.0904	0.9253	
Perceived Benefits	0.0546	−0.0066	−0.8895	0.8915

Notes: Diagonal entries are the square roots of AVE, and lower-triangular entries are inter-construct correlations.

**Table 5:** Baseline-adjusted effects of the three public opinion conditions on post-exposure perceived benefits, perceived sacrifices, and purchase intention (N = 742).

Outcome/Contrast	Estimate	95% CI	Holm *p*	Omnibus and Adjusted-Mean Summary
Perceived benefits: omnibus	—	—	<0.001	F(2, 705) = 918.06; partial η^2^ = 0.723; means N/P/B = 2.17/4.53/4.29
Positive–negative	2.367	[2.233, 2.501]	<0.001	—
Balanced–negative	2.125	[1.980, 2.270]	<0.001	—
Positive–balanced	0.242	[0.139, 0.344]	<0.001	—
Perceived sacrifices: omnibus	—	—	<0.001	F(2, 705) = 1222.02; partial η^2^ = 0.776; means N/P/B = 4.34/1.67/2.45
Positive–negative	−2.666	[−2.796, −2.537]	<0.001	—
Balanced–negative	−1.885	[−2.061, −1.709]	<0.001	—
Positive–balanced	−0.781	[−0.943, −0.619]	<0.001	—
Purchase intention: omnibus	—	—	<0.001	F(2, 705) = 820.30; partial η^2^ = 0.699; means N/P/B = 1.86/4.45/3.97
Positive–negative	2.587	[2.428, 2.746]	<0.001	—
Balanced–negative	2.107	[1.939, 2.274]	<0.001	—
Positive–balanced	0.480	[0.322, 0.638]	<0.001	—

Note: Estimates were obtained from baseline-adjusted ANCOVA models with HC3 standard errors. Each model included the corresponding pre-exposure outcome. The negative-content condition was the omitted active reference category. N, P, and B denote the negative, positive, and balanced conditions, respectively. Covariates included gender, age, education, occupation, annual income, household composition, primary purchase channel, consumption frequency, residential proximity to water bodies, self-rated health status, and prior experience with aquatic product safety incidents. Pairwise comparisons were adjusted using the Holm procedure. Partial η^2^ is reported as the omnibus effect size.

**Table 6:** Parallel mediation of randomized public opinion condition contrasts on purchase intention through perceived benefits and perceived sacrifices (N = 742).

Condition Contrast/Effect	Estimate	Bootstrap SE	95% Bootstrap CI
Positive vs. negative: via benefits	0.823	0.128	[0.579, 1.081]
Positive vs. negative: via sacrifices	1.016	0.147	[0.738, 1.311]
Positive vs. negative: total indirect	1.839	0.132	[1.579, 2.096]
Positive vs. negative: direct	0.752	0.142	[0.488, 1.043]
Positive vs. negative: total	2.592	0.063	[2.472, 2.717]
Balanced vs. negative: via benefits	0.739	0.113	[0.523, 0.964]
Balanced vs. negative: via sacrifices	0.719	0.106	[0.518, 0.933]
Balanced vs. negative: total indirect	1.458	0.102	[1.259, 1.657]
Balanced vs. negative: direct	0.645	0.125	[0.409, 0.897]
Balanced vs. negative: total	2.103	0.068	[1.970, 2.236]
Difference between paths: positive	−0.193	0.239	[−0.673, 0.261]
Difference between paths: balanced	0.020	0.190	[−0.364, 0.381]

Note: Estimates were obtained from a simultaneous parallel-mediation model using 5000 nonparametric bootstrap resamples (N = 742). Positive and balanced-information conditions were compared with the active negative-content reference condition. The model adjusted for baseline purchase intention, baseline perceived benefits, baseline perceived sacrifices, gender, age, education, occupation, annual income, household composition, primary purchase channel, consumption frequency, residential proximity to water bodies, self-rated health status, and prior experience with aquatic product safety incidents. A 95% bootstrap confidence interval excluding zero indicates statistical evidence of an indirect effect.

**Table 7:** Baseline-adjusted OLS estimates of regulatory-focus moderation of post-exposure perceived benefits, perceived sacrifices, and purchase intention (N = 742).

Variables	(2)	(3)	(4)
	Perceived Benefits	Perceived Sacrifices	Purchase Intention
Positive	2.368 ***	−2.698 ***	2.594 ***
	(0.054)	(0.054)	(0.067)
Balanced	2.128 ***	−1.877 ***	2.106 ***
	(0.059)	(0.071)	(0.069)
Promotion Focus	−0.249 **	0.101	−0.091
	(0.081)	(0.075)	(0.077)
Prevention Focus	−0.165	−0.043	−0.147
	(0.092)	(0.062)	(0.079)
Positive × Promotion Focus	0.273 *	−0.131	0.162
	(0.092)	(0.103)	(0.104)
Balanced × Promotion Focus	0.320 **	−0.599 ***	0.226
	(0.098)	(0.129)	(0.118)
Positive × Prevention Focus	0.221	−0.033	0.180
	(0.101)	(0.099)	(0.105)
Balanced × Prevention Focus	0.217	−0.027	0.278 *
	(0.103)	(0.118)	(0.114)
Corresponding baseline outcome	YES	YES	YES
Control variables	YES	YES	YES
Constant	0.570	4.439 ***	0.745
	(0.473)	(0.371)	(0.469)
AdjR^2^	0.8075	0.7602	0.7384
Full-model robust F(42, 699)	80.81 ***	104.88 ***	68.61 ***
Joint-interaction robust F(4, 699)	6.26 ***	6.09 ***	3.67 **

Note: Positive and balanced are condition indicators; the negative-content condition is the active reference. Promotion-focus and prevention-focus scores were mean-centered and entered together. Each model adjusts for the corresponding baseline outcome and for gender, age, education, occupation, annual income, household composition, primary purchase channel, consumption frequency, residential proximity to water bodies, self-rated health status, and prior experience with aquatic product safety incidents. HC3 heteroskedasticity-robust standard errors are reported in parentheses. For the 12 interaction coefficients, significance stars are based on Benjamini–Hochberg FDR-adjusted *p* values (q values); stars for other coefficients and model tests are based on unadjusted HC3 *p* values. * *p* or q < 0.05, ** *p* or q < 0.01, and *** *p* or q < 0.001.

## Data Availability

The data presented in this study are available on request from the corresponding authors. The data are not publicly available now because the Central Public-Interest Scientific Institution Basal Research Fund [grant number: 2026A006] is not yet closed.
